# Fine-tuning the ubiquitin code at DNA double-strand breaks: deubiquitinating enzymes at work

**DOI:** 10.3389/fgene.2015.00282

**Published:** 2015-09-08

**Authors:** Elisabetta Citterio

**Affiliations:** Division of Molecular Genetics, Netherlands Cancer Institute, AmsterdamNetherlands

**Keywords:** ubiquitin, DNA double-strand breaks (DSBs), deubiquitinating enzymes (DUBs), histone H2A, chromatin, DNA damage response (DDR), hematopoietic stem cell (HSC), cancer

## Abstract

Ubiquitination is a reversible protein modification broadly implicated in cellular functions. Signaling processes mediated by ubiquitin (ub) are crucial for the cellular response to DNA double-strand breaks (DSBs), one of the most dangerous types of DNA lesions. In particular, the DSB response critically relies on active ubiquitination by the RNF8 and RNF168 ub ligases at the chromatin, which is essential for proper DSB signaling and repair. How this pathway is fine-tuned and what the functional consequences are of its deregulation for genome integrity and tissue homeostasis are subject of intense investigation. One important regulatory mechanism is by reversal of substrate ubiquitination through the activity of specific deubiquitinating enzymes (DUBs), as supported by the implication of a growing number of DUBs in DNA damage response processes. Here, we discuss the current knowledge of how ub-mediated signaling at DSBs is controlled by DUBs, with main focus on DUBs targeting histone H2A and on their recent implication in stem cell biology and cancer.

## Introduction

The ability of cells to maintain the integrity of their genome is crucial for organism physiology, including stem cell and tissue homeostasis and cancer avoidance (Jackson and Bartek, 2009; Blanpain et al., 2011; Behrens et al., 2014). A complex surveillance network protects cells from the continuous threat of exogenous as well as endogenously generated genotoxic insults. This includes multiple pathways, collectively called the DNA damage response (DDR), which ensure effective DNA damage detection, signaling, and repair (Ciccia and Elledge, 2010). DDR coordinates DNA repair with vital cellular functions, including transcription and DNA replication, and determines the fate of the cell after DNA damage. Post-translational modification of proteins by the 76 amino acid protein ubiquitin (ub) plays a central role in various aspects of DDR (Lukas et al., 2011; Jackson and Durocher, 2013). ub is conjugated to lysine residues (Lys, K) in target proteins through the activities of E1, E2, and E3 enzymes (Komander and Rape, 2012). Proteins can be modified with one ub moiety (mono-ubiquitination) or with polymeric ub chains. The use of one of the seven lysines of ub (K6, K11, K27, K29, K33, K48, and K63) or the N-terminal amine (“linear”) for chain formation allows diverse conformations, conferring a great signaling potential to the ub system. The resulting ub “code” can target the substrate for degradation, or regulate its interactions, localization or activity. Typically, K48-linked ub chains serve as proteasomal degradation signals, while K63-linked chains (K63-ub) are non-degradative. The ub code is ultimately “read” by proteins endowed with ub binding domains, which determine distinct outcomes in the cell (Komander and Rape, 2012).

Modification by ub is regulated by the catalytic activities of deubiquitinating enzymes (DUBs; also known as deubiquitinases or deubiquitylating enzymes), which can cleave ub from proteins or process all types of ub-chains (Komander et al., 2009). The human genome encodes for ≈90 potential DUBs, many of which are strongly implicated in cancer and other pathologies, including neurodegenerative, hematological and infectious diseases (Nijman et al., 2005; Komander et al., 2009). DUBs can be subdivided in five subfamilies, four belonging to the cysteine proteases group [ubiquitin-specific proteases (USPs), ovarian tumor proteases (OTUs), ubiquitin C-terminal hydrolases (UCHs) and Josephin domain DUBs], and one consisting of the JAMM/MPN+ family of metalloproteases (Komander et al., 2009). One key function of DUBs is the generation of free ub from ub precursors. Second, by cleaving mono-ub adducts or poly-ub chains from target proteins, DUBs can reverse a non-degradative ub signal or stabilize target proteins by rescuing them from proteasomal or lysosomal degradation. Third, DUBs can cleave the isopeptide bond from within the ub polymer, an activity that allows editing ub chains and thereby ub-mediated signal (Komander and Rape, 2012; Heride et al., 2014). Beside the catalytic domain, DUBs contain multiple domains, some of which aid in substrate or in protein–protein interactions, endowing the DUBs with the ability to display specificity at the protein substrate level as well as at the level of ub chain types (Komander and Rape, 2012).

It has become clear that reversal of substrate ubiquitination by DUBs is a critical regulatory mechanism throughout the DDR. In this review, I will focus on recent discoveries involving DUBs in the modulation of ub-mediated DDR at DNA double-strand breaks (DSBs), with emphasis on histone H2A targeting DUBs. Readers are referred to recent reviews for a more comprehensive overview of DUB functions (Komander et al., 2009; Clague et al., 2012; Belle and Nijnik, 2014; Sahtoe and Sixma, 2015), ub and DUBs roles in DDR (Ulrich, 2012; Jackson and Durocher, 2013; Jacq et al., 2013; Brown and Jackson, 2015) or for detailed information regarding the ubiquitination/deubiquitination process (Komander and Rape, 2012; Heride et al., 2014).

## Ubiquitin-Mediated DDR Signaling at DNA Double-Strand Breaks

**FIGURE 1:**
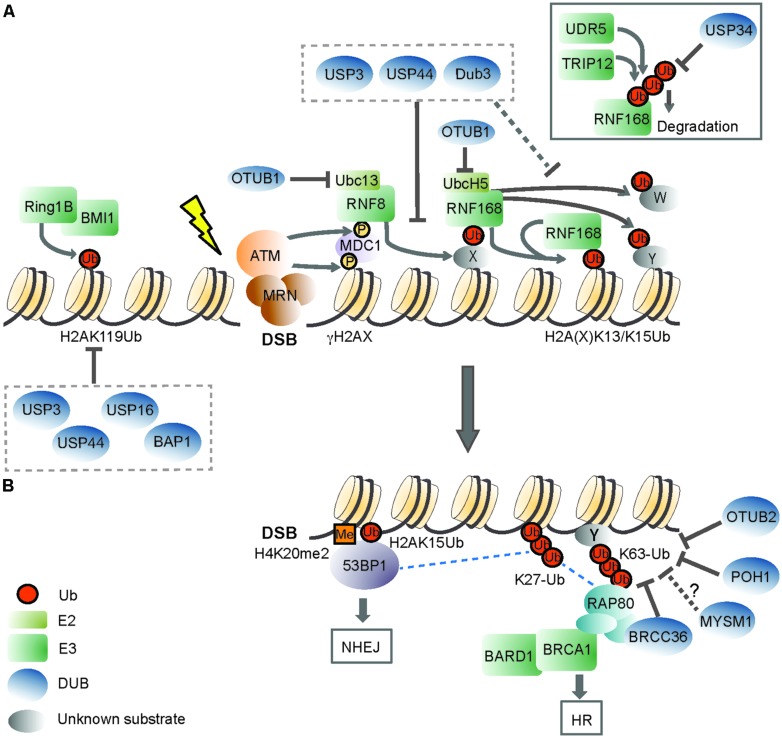
**The role of deubiquitinating enzymes in the chromatin-based response to DNA double-strand breaks. (A)** Recognition of a DNA double-strand break (DSB) by the MRN (MRE11-RAD50-NBS1) complex initiates DDR signaling, triggering ATM (ataxia-telangectasia mutated) kinase-dependent phosphorylation of H2AX (γH2AX). ATM phosphorylates also MDC1 (mediator of DNA damage checkpoint protein 1), which is recognized by the RING finger 8 (RNF8) E3 ligase. The activity of RNF8 is required for recruitment of a second E3, RNF168. RNF168 mono-ubiquitinates H2A-type histones on Lys13 and Lys15 (H2A(X)K13/K15Ub), and the concerted action of RNF8/RNF168 leads to the formation of K63-linked ubiquitin (Ub) chains on these lysines and to ubiquitination of other substrates (Y and W). RNF168 can bind to its own products, thereby amplifying chromatin ubiquitination around the DSB. OTUB1 opposes RNF168 activity in a non-catalytic manner, by binding to the E2 ubiquitin-conjugating enzymes UBC13 and UbcH5. USP3, USP44, and Dub3 DUB activities impair RNF168 recruitment, suggesting that they can target RNF8 substrate(s). These DUBs may also cleave RNF168-mediated ubiquitinated H2A(X). Excessive RNF168-dependent chromatin ubiquitination is limited by the TRIP12 and UBR5 E3 ligases, which target RNF168 for proteasomal degradation. USP34, instead, counteracts DSB-induced RNF168 ubiquitination. DSBs also trigger the recruitment of the Polycomb group E3 RING1B/BMI1, which mono-ubiquitinates H2A on Lys119 (H2AK119Ub) to locally repress transcription. USP16 and BAP1 target the H2AK119Ub mark, and USP16 activity is required for re-activation of DSB-induced transcriptional silencing. USP3 and USP44 oppose to steady-state mono-ubiquitinated H2A, which is primarily constituted by H2AK119Ub. **(B)** DSB-induced ubiquitin signals are recognized by downstream DDR factors. 53BP1 (p53 binding protein 1) recognizes the H2AK15Ub mark by its UDR (ubiquitination-dependent recruitment) motif, and dimethylated H4K20 (H4K20me2) with its TUDOR domain. One way BRCA1 (Breast cancer 1) is recruited to DSBs is through its interaction with RAP80 (receptor-associated protein 80), a protein that effectively binds K63-ub conjugates. BRCA1 has E3 ligase activity and functions as a heterodimer with BARD1 (BRCA1-associated RING domain protein 1). The DUBs BRCC36, POH1, and OTUB2 regulate DDR signaling by hydrolyzing DSB-induced K63-ub chains. OTUB2 also opposes to RNF8-dependent ubiquitination of L3MBTL1 (not shown). Whether MYSM1, which also possesses K63-ub cleavage activity, participates in this step of DDR is an open question. RNF168-mediated K27-linked ub chains on H2A/H2AX constitute additional DDR signals, and BRCA1/BARD1 catalyzed K6-linked chains (not shown) may also contribute to DDR. Specific activities that oppose to these atypical ub chains in DDR are not known. 53BP1 and BRCA1 determine effective DSB repair, with 53BP1 committing to NHEJ (non-homologous end joining) and BRCA promoting HR (homologous recombination). Dashed lines indicate proposed protein–protein interactions. X indicates an unknown RNF8 substrate(s), and Y and W indicate unknown RNF168 substrates. Please refer to the main text for details.

In eukaryotic cells, the packaging of DNA with histone proteins into chromatin, the basic unit being the nucleosome, has major impact on DNA damage signaling and repair. This is because on one hand the compact organization of chromatin intrinsically limits the degree of access to DNA. At the same time, however, chromatin provides a sensitive regulatory platform for DDR through post-translational modifications to both chromatin components (i.e., histones) and non-chromatin proteins (Lukas et al., 2011). Non-proteolytic (mono-) ubiquitination of histones is a prevalent modification in mammalian cells (Goldknopf and Busch, 1977). Work from several groups has shown that histone ubiquitination at the chromatin surrounding DSBs is a key step in DDR activation (Lukas et al., 2011; **Figure 1**). Phosphorylation of the histone variant H2AX (yielding γH2AX) by the ataxia telangectasia mutated (ATM) checkpoint kinase promotes the binding of the E3 ligase RNF8 through the mediator protein MDC1 to damage sites, where it initiates ub signaling (Huen et al., 2007; Kolas et al., 2007; Mailand et al., 2007; Wang and Elledge, 2007). A second E3, RNF168, is then recruited through RNF8-ubiquitinated substrates to the proximity of the lesion and starts catalysis of H2A/H2AX ubiquitination on Lys 13 (H2AK13ub) and/or Lys15 (H2AK15ub; Gatti et al., 2012; Mattiroli et al., 2012). The coordinated activities of RNF8/RNF168 with HERC2 and the E2 Ubc13 lead to the formation on H2A/H2AX of K63-ub, a prevalent ub linkage at DSBs (Doil et al., 2009; Pinato et al., 2009; Stewart et al., 2009; Gatti et al., 2012; Mattiroli et al., 2012). A major outcome of RNF8/RNF168-mediated ubiquitination is recruitment/stable accumulation of DDR proteins at the lesion, with the tumor suppressors BRCA1 (breast cancer 1, early onset) and 53BP1 (p53 binding protein 1) representing the two key effectors of the pathway (Lukas et al., 2011). Importantly, the interplay between BRCA1 and 53BP1 determines effective DSB repair by one of the two major DSB repair pathways, with BRCA1 promoting the error-free homologous recombination (HR) process while 53BP1 committing to non-homologous end joining (NHEJ; Cao et al., 2009; Bouwman et al., 2010; Bunting et al., 2010). By influencing the relative kinetics of these DDR effectors at DSBs, the RNF8 pathway is functionally implicated in determining the repair pathway choice, which is critical to genome maintenance (Panier and Boulton, 2014).

53BP1 recognizes the DSB-specific H2AK13/15ub mark through its UDR (ubiquitination-dependent recruitment) motif, which, together with the Tudor domain reads a bivalent ubiquitination-methylation signal at damage sites (Fradet-Turcotte et al., 2013). While no ub-binding domains have been yet identified in BRCA1, it is clear that BRCA1 stable accumulation at the DSB-flanking chromatin is dependent on its interaction with RAP80, a protein that binds K63-linked ub conjugates through its tandem ub-interacting motifs (Huen et al., 2007; Kolas et al., 2007; Mailand et al., 2007; Wang et al., 2007). Initial BRCA1 recruitment, however, appears independent of Rap80 (Hu et al., 2011; Yin et al., 2012) and involves binding of a small fraction of BRCA1 directly at the DNA break through interaction with the DSB recognition factor Nbs1 (Goldstein and Kastan, 2015).

Signaling through chromatin ub during DDR is multifaceted, engaging several E3 ligases and modification by ub and ub-like proteins of (non-)histone proteins (Lukas et al., 2011; Jackson and Durocher, 2013; Brown and Jackson, 2015). One of these E3 is the PcG transcriptional repressor complex RING1b/BMI1 (Chou et al., 2010; Ismail et al., 2010; Gieni et al., 2011; Ginjala et al., 2011; Vissers et al., 2012). RING1b/BMI1 is part of the polycomb repressive complex 1 (PRC1) and is responsible for the mono-ubiquitination of H2A at the canonical site, Lys-K119 (yielding H2AK119ub), a histone mark that constitutes about 10–15% of H2A under physiological conditions (Goldknopf and Busch, 1977; Wang et al., 2004; Di Croce and Helin, 2013). While the major source of DSB-induced H2A poly-ubiquitination appears to be RNF8/RNF168-dependent (Lukas et al., 2011; Mattiroli et al., 2012), RING1b/BMI1 is required for silencing transcription in response to neighboring DSBs through local enhancement of H2A/H2AXK119ub (Ismail et al., 2010; Ginjala et al., 2011; Kakarougkas et al., 2014; Ui et al., 2015). RING1b/BMI1 is also thought to contribute to maintenance of active ATM at DSBs (Pan et al., 2011; Wu et al., 2011a). Interestingly, both ubiquitination of H2AK13/15ub by RNF168 and H2AK119ub by RING1b/BMI1 depend on an intact nucleosome acidic patch (Leung et al., 2014). It is proposed that this structure within H2A/H2AX serves as a scaffold to integrate differential signals on H2A (Leung et al., 2014). In contrast, the functional significance of H2AK127-129ub by the E3 BRCA1/BARD1 is still unclear (Kalb et al., 2014).

ATM-dependent mono-ubiquitination of histone H2B (H2BK120ub) also occurs in response to DSBs (Moyal et al., 2011; Nakamura et al., 2011). Similarly to H2A, H2B mono-ubiquitination fulfills a dual function, in gene transcription and in DDR (Shiloh et al., 2011). However, unlike H2AK119ub, H2BK120ub has been mostly linked to gene activation, in part through chromatin decompaction at transcribed regions (Shiloh et al., 2011; Fuchs and Oren, 2014). Transcriptional silencing and chromatin decondensation are both critical to ensure proper DNA repair. Given the physical proximity between H2BK120ub and H2AK13/15ub within the nucleosome, it is possible that these marks cooperate in the integration of these processes (Shiloh et al., 2011; Mattiroli et al., 2012; Jackson and Durocher, 2013). To this end, it is relevant the finding that RNF8 synergizes with its structurally related kinase CHFR (checkpoint with fork-head associated and ring finger) in H2A as well as H2B ubiquitination, an activity that promotes chromatin relaxation, ATM activation and genome stability in mice (Wu et al., 2011b).

DNA damage signaling and activation of DNA damage checkpoints result in transient cell-cycle arrest or permanent cell cycle withdrawal (Ciccia and Elledge, 2010). An important, yet not well-understood, aspect of ub-based DDR is how spatio-temporal control of the signaling is achieved. In particular, it remains to clarify in full how disassembly of the repair complexes occurs after the damage has been repaired, which enzymes are involved and how this impacts on termination of the DNA damage checkpoint, and ultimately on genomic stability and survival. Notably, excessive chromatin ub at DSBs associates with enhanced repair and spreading of gene silencing beyond the physiological boundaries, underscoring the relevance of tight control of this pathway (Gudjonsson et al., 2012). Consistently, several mechanisms have been discovered which modulate the magnitude of RNF8/RNF168-dependent ubiquitination, and regulatory inhibition has emerged as an integral DDR component (Panier and Durocher, 2013). Examples of such mechanisms include proteolytic ubiquitination of RNF168 (Gudjonsson et al., 2012), competition for recruitment of RNF168-dependent effectors by a RNF168 paralog (Panier et al., 2012; Poulsen et al., 2012), eviction of ubiquitinated proteins (Acs et al., 2011) and, as reviewed here, DUBs activities at multiple levels of the RNF8/RNF168 ubiquitination cascade.

## Modulation of DSB Signaling by DUBs

Less then a decade ago, studies on the DUBs BRCC36, USP3 and USP16 first uncovered the importance of DUB activity in the modulation of ub-based DDR by regulating recruitment of RNF168-responsive factors, checkpoint recovery, and DSB-induced transcriptional silencing, respectively (Nicassio et al., 2007; Sobhian et al., 2007; Wang and Elledge, 2007; Doil et al., 2009; Shao et al., 2009; Shanbhag et al., 2010). Since then, an increasing body of evidence supports the reversal of DNA damage-induced chromatin ubiquitination by DUBs as a key aspect of the DSB response (Jackson and Durocher, 2013; Panier and Durocher, 2013). Indeed, siRNA-based screens in mammalian cells recently revealed that a great part of the DUB family has functional connections with the DSB response (Kato et al., 2014; Nishi et al., 2014; Yu et al., 2014) and a number of DUBs have been implicated as regulatory components of the RNF8 pathway (**Table 1** and **Figure 1**). Recent findings have broadened the contribution of DUBs targeting histone H2A, the critical RNF8-RNF168 substrate, to DDR, and will be the main focus of the next section.

**Table 1:** Mammalian DUBs that have been associated with the chromatin-based DSB response and their implications in organism physiology and human disease.

DUB	Substrate(s)	Proposed role(s) in DDR	Other cellular functions	Implications in organism physiology/disease	Reference
**Ubiquitin specific proteases (USP)**					
USP3	H2A, H2AX, H2B, RIG1	Antagonizes RNF168 IRIFs; promotes, DSB repair, sensitization:IR^1^	Cell cycle; type I interferon signaling; HGF-dependent scattering response	*Usp3*-null mice display lymphopenia, decline in HSC function and spontaneous tumorigenesis upon aging	Nicassio et al. (2007), Buus et al. (2009), Doil et al. (2009), Mosbech et al. (2013), Cui et al. (2014), Lancini et al. (2014), Nishi et al. (2014), Sharma et al. (2014)
USP16/Ubp-M	H2A^2^	DSB-induced gene silencing	Cell cycle; transcription; ESC differentiation	*Usp16* knockout is embryonic lethal. Trisomy of *Usp16* (Ts65Dn model for Down’s syndrome) associates with reduced HSC self-renewal	Joo et al. (2007), Shanbhag et al. (2010), Adorno et al. (2013), Yang et al. (2014), Zhang et al. (2014)
Dub3/USP17L2	H2AX, Cdc25A	Antagonizes RNF168 IRIFs	Cell cycle/DNA damage checkpoint; ESC self-renewal	Promotes growth of human breast cancer xenograft tumors	Pereg et al. (2010), van der Laan et al. (2013), Delgado-Diaz et al. (2014)
USP34	RNF168, Axin	Stabilizes RNF168 protein	Wnt/beta-catenin signaling	n.d.	Lui et al. (2011), Sy et al. (2013)
USP44	H2A, H2B, CDC20	Antagonizes RNF168 and 53BP1 IRIFs	Spindle assembly checkpoint; prevents aneuploidy	*Usp44*-null mice develop spontaneous tumors, in particular in the lung	Stegmeier et al. (2007), Song et al. (2010), Zhang et al. (2011, 2012), Fuchs et al. (2012), Mosbech et al. (2013)
**Ubiquitin C-terminal hydrolases (UCH)**					
BAP1	H2AK119ubH2AX	Promotes DSB repair. Sensitization: IR and PARP inhibitors	Cell cycle progression; transcription	*Bap1*-knockout is embryonic lethal; *Bap1* deletion in adulthood results in HSC defects and myeloid transformation. Human tumor suppressor	Nishikawa et al. (2009), Harbour et al. (2010), Scheuermann et al. (2010), Bott et al. (2011), Dey et al. (2012), Peña-Llopis et al. (2012), Carbone et al. (2013), Ismail et al. (2014), Nishi et al. (2014), Yu et al. (2014)
**JAMM/MPM+ metallo-proteases**					
BRCC36, (BRCC3)	K63-ub, H2A-K63-ub, IFNAR1	Restrict DNA end resection; limits HR. Sensitization: IR	Interferon responses	Aberrant expression in human breast tumors	Dong et al. (2003), Sobhian et al. (2007), Wang and Elledge (2007), Cooper et al. (2009), Shao et al. (2009), Coleman and Greenberg (2011), Hu et al. (2011), Zheng et al. (2013)
MYSM1/2A-DUB	H2A	Promotes DSB repair. Sensitization:IR^1^	Transcription	*Mysm1*-null mice display partial embryonic lethality, growth retardation, epidermal abnormalities, multi-lineage hematopoietic defects, HSC deficiencies and predisposition to lymphoma	Zhu et al. (2007), Jiang et al. (2011), Nijnik et al. (2012), Nandakumar et al. (2013), Wang et al. (2013), DiTommaso et al. (2014), Liakath-Ali et al. (2014), Nishi et al. (2014), Won et al. (2014), Belle et al. (2015), Gatzka et al. (2015)
POH1/PSMD14	K63-ub	Restricts 53BP1; promotes RAD51; sensitization: IR, *cis*-platin, HU	Proteasome activity	n.d.	Yao and Cohen (2002), Butler et al. (2012), Kakarougkas et al. (2013)
**Ovarian tumor proteases (OTU)**					
OTUB1	K48-ub	Non-catalytical inhibition of RNF168 and of K63-ub-chains. Sensitization:IR	p53 stability; TGFβ signaling; c-IAP1 stability	n.d.	Nakada et al. (2010), Juang et al. (2012), Sun et al. (2012), Wiener et al. (2012), Herhaus et al. (2013), Mevissen et al. (2013)
OTUB2	L3MBTL1, K63-ub^3^	Suppresses HR, sensitization:NCS and CPT	n.d.	n.d.	Kato et al. (2014)

*All DUBs discussed here show certain degree of evolutionary conservation. To date, their role in DDR emerged from studies in the mammalian system or chicken DT40 cells (Yu et al., 2014). All DUBs, except for OTUB2, show sequence conservation in *Xenopus laevis* and in zebrafish (*Danio rerio*; www.ensembl.org; Tse et al., 2009). *Drosophila melanogaster* orthologs of BAP1 (Calypso; Scheuermann et al., 2010), PHO1/PSMD1 (Rpn11) and USP34 (puf/puffyeye) have been described (www.ensembl.org; Tsou et al., 2012). PHO1/PSMD1 has an ortholog in *Saccharomyces cerevisiae*, RPN11. The *S. cerevisiae* DUB closest to USP3, USP16, and USP44 is Ubp8 (www.ensembl.org; Sloper-Mould et al., 1999), which targets yeast H2B (Henry et al., 2003). c-IAP1, cellular inhibitor of apoptosis 1; CTP, camptothecin; ESC, embryonic stem cell; HGF, hepatocyte growth factor; HR, homologous recombination; HSC, hematopoietic stem cell; HU, hydroxyurea; IR, ionizing radiation; H2A-K63-ub, H2A modified by lysine 63-linked ub chains; IRIFs, ionizing radiation induced foci; NCS, neocarzinostatin radiomimetic drug; PARP, poly(ADP-ribose) polymerase; IFNAR1, type 1 interferon (IFN) receptor chain 1; K48-ub, lysine 48-linked ub chains; K63-ub, lysine 63-linked ub chains DNA damage-inducing agents known to sensitize (tumor) cells depleted for the DUB or ^1^DUB knockout mice are indicated; ^2^not H2B, not K63-ub; ^3^not H2A-K63-ub. n.d., not determined.*

### USP3

The ub-specific protease USP3 is a chromatin-associated protein endowed with the ability of binding to ubiquitinated H2A through its Zn finger UBP domain (Nicassio et al., 2007). Biochemical and cellular studies showed that both uH2A and uH2B are relevant USP3 targets (Nicassio et al., 2007; Mosbech et al., 2013). We recently found that deletion of *Usp*3 in mice leads to a measurable increase in uH2A and uH2B in freshly isolated tissues, suggesting that USP3 has a non-redundant role in preventing accumulation of uH2A and uH2B *in vivo* (Lancini et al., 2014). USP3 is implicated in the regulation of S-phase progression (Nicassio et al., 2007), a role that may be relevant also in the context of HSC biology (see below; Lancini et al., 2014), and in the DSB response. Removal of the ub mark by USP3 appears important for the prevention and/or for proper repair of spontaneous DNA damage as well as for DSB repair and resolution of DDR signaling upon clastogen-induced DSBs (Nicassio et al., 2007; Lancini et al., 2014; Nishi et al., 2014). In line with a role for USP3 in the DSB response, *Usp3*-deleted HSCs and mice are hypersensitive to IR (Lancini et al., 2014). Mechanistically, the data suggest that USP3 limits the RNF8/RNF168 pathway by reversing the ubiquitination catalyzed by these ligases. In fact, ectopic expression of wild-type USP3, but not a catalytically inactive mutant protein, prevented accumulation of RNF168 and 53BP1 at IR-induced DNA damage foci (IRIFs), while leaving upstream signaling through γH2AX and RNF8 intact (Doil et al., 2009; Mosbech et al., 2013). Notably, RNF8 also ubiquitinates non-histone proteins at DSBs (Acs et al., 2011; Meerang et al., 2011; Mallette et al., 2012) and RNF8-catalyzed ub conjugates distinct form H2A/H2AX are required for RNF168 recruitment (Mattiroli et al., 2012). Given the excess of chromatin-bound ub conjugates compared to the increase of uH2A measured upon USP3 loss (Nicassio et al., 2007; Lancini et al., 2014), it is conceivable that modulation of DDR signaling by USP3 may involve deubiquitination of additional, non-histone, targets. Because of spatial proximity, it is possible that these other USP3 targets may include as-yet undefined RNF8 substrates and/or DDR factors, many of which are known to be ubiquitinated, including RNF168 and 53BP1 (Gudjonsson et al., 2012; Jackson and Durocher, 2013). In this regard, it is of note that ectopic green fluorescent protein (GFP)-USP3 fusion protein could not be detected at DNA damage foci (Mosbech et al., 2013). While this may reflect detection limitations liked to the experimental conditions, it is also possible that USP3 may, at least in part, act in DDR through indirect mechanisms.

### USP16

By a conventional chromatography approach, USP16 (also known as Ubp-M) was purified from HeLa cells as an enzyme strongly active in removing the mono-ub moiety form H2A (Joo et al., 2007). Consistently, growing evidence support a functional role of USP16 in counteracting PcG-mediated gene silencing through H2AK119ub deubiquitination (Joo et al., 2007; Adorno et al., 2013; Frangini et al., 2013; Yang et al., 2014). Local transcription inhibition also occurs in *cis* at the chromatin contiguous to sites of DSB induction, a phenomenon dependent on the ATM/RNF8/RNF168 ub pathway (Shanbhag et al., 2010) and on RING1b/BMI1 (Kakarougkas et al., 2014; Ui et al., 2015). Notably, siRNA-mediated knock- down of *Usp16* expression resulted in sustained DSB-induced uH2A, which prolonged gene silencing in the proximity of the DSB (Shanbhag et al., 2010). These findings implicate USP16 in controlling reversible transcriptional inhibition at DSBs by removal of uH2A and place USP16 at the interplay between DDR signaling and local gene silencing (Shanbhag et al., 2010). Whether USP16 impacts on DSB repair is unclear. In fact, USP16 was not readily detected at IRIFs and its ectopic expression failed to limit 53BP1 recruitment at DSBs (Mosbech et al., 2013; Nishi et al., 2014). Also, USP16 did not score above significance in a siRNA-based *in vitro* functional screen for DUBs affecting spontaneous DNA damage, DSB repair or DSB-induced G2/M checkpoint (Nishi et al., 2014). Recently, USP16 has been found to interact with HERC2 (Sowa et al., 2009; Zhang et al., 2014), an E3 ligase known to stabilize the RNF8 interaction with its cognate E2 ubc13 and to promote DSB signaling (Bekker-Jensen et al., 2010). This finding puts forward a potential mechanism through which USP16 may be implicated in the RNF8 pathway.

### USP44

Functional genetic screens first uncovered USP44 as a regulator of the mitotic spindle assembly checkpoint through deubiquitination of the APC activating subunit CDC20 (Stegmeier et al., 2007; Song et al., 2010). Further establishing USP44 function during mitosis, a genetic approach revealed the requirement of USP44 for proper centrosome separation and positioning, ensuring accurate chromosome segregation (Zhang et al., 2012). More recently, an overexpression screen for DUBs antagonizing the RNF8-RNF168 pathway was performed in human U2OS cells. Among the 60 DUBs tested, USP44 was identified as one of the five DUBs that potently abrogate recruitment of 53BP1 to IRIFs, along with the previously identified USP3 (Nicassio et al., 2007; Doil et al., 2009) and OTUB1 (Nakada et al., 2010; Mosbech et al., 2013). Consistent with such an activity, GFP-USP44 re-localized to DNA damage sites generated by laser micro-irradiation, in a manner dependent on RNF8 and RNF168 (Mosbech et al., 2013; Nishi et al., 2014). Direct reversal of H2A ubiquitination is likely one of the inhibitory mechanisms through which USP44 opposes to 53BP1 recruitment. In fact, inducible expression of wild type USP44 was accompanied by: (i) strong reduction of cellular uH2A, indicating that USP44 can target the most abundant H2AK119ub mark; (ii) reduction of mono- di- and tri-ub H2A upon H2A co-overexpression, a condition that allows visualization of poly-ub chains; (iii) displacement of endogenous RNF168 from IRIFs. USP44 displays certain specificity for histones and can target, besides uH2A, uH2B (Mosbech et al., 2013), an activity that may also contribute to DDR (Shiloh et al., 2011). These findings are in line with USP44 being a chromatin-associated protein (Stegmeier et al., 2007; Fuchs et al., 2012) and with the identification of H2A and H2B as significantly enriched interactors in a proteomics survey (Sowa et al., 2009).

Notably, USP44 shares with USP3 the ability to target both uH2A and uH2B and to displace RNF168 and 53BP1 from IRIFs (Nicassio et al., 2007; Doil et al., 2009; Fuchs et al., 2012; Mosbech et al., 2013). This suggests that these DUBs may have related/overlapping functions in DDR, a hypothesis also supported by their clustering in phylogenetic analysis of DUBs based on DSB repair activity (Nishi et al., 2014). One could envisage temporal or spatial regulation of the activities of these DUBs in relation to different chromatin territories at the DSB (Chapman et al., 2012; Kakarougkas et al., 2013), cell cycle or chromatin states. It is currently not known if their effects on uH2A/uH2B are additive. USP3 knock-down alone causes a significant, although modest, enlargement of spontaneous and IR induced 53BP1 foci (Nicassio et al., 2007; Gudjonsson et al., 2012). The engagement of USP3 and USP44 potentially similar activities at DSBs may reflect the need to effectively control RNF168-mediated signaling. It will be interesting to address this interplay and to investigate how USP44 impacts on genomic stability and cellular survival upon damage.

### BAP1

The BRCA1-associated protein 1 (BAP1; also known as UCHL2) displays DUB activity for H2AK119ub (Scheuermann et al., 2010). BAP1 is active in the context of the PcG repressive complex PR-DUB, an evolutionary conserved complex comprising BAP1 and additional sex combs-like 1 (ASXL1) in mammals, and their orthologs Calypso and Asx in *Drosophila* (Scheuermann et al., 2010). The *Drosophila* PR-DUB complex is thought to maintain a balanced H2AK119ub status required for proper HOX gene silencing (Scheuermann et al., 2010). Consistent with functional conservation, BAP1 interaction with ASLX1 and functional implication in PcG target genes regulation are documented in mammalian cells (Bott et al., 2011; Dey et al., 2012; Peña-Llopis et al., 2012). In addition, a number of studies implicate BAP1 in the DSB response. Indeed, shRNA-mediated silencing/loss of *Bap1* results in defective DSB repair (Nishi et al., 2014; Yu et al., 2014) and *Bap1*-null cancer cell-lines are hypersensitive to IR (Nishikawa et al., 2009; Bott et al., 2011; Peña-Llopis et al., 2012; Ismail et al., 2014; Yu et al., 2014). Experimental evidence of chromatin recruitment based on chromatin immunoprecipitation (ChIP) at I-Sce-induced breaks (Yu et al., 2014) and visualization by microscopy at sites of micro-irradiation (Ismail et al., 2014; Nishi et al., 2014) suggests that BAP1 function is exerted at the chromatin flanking DBSs. In fact, enhancement of uH2A/uH2AX levels upon IR was reported upon BAP1 depletion (Ismail et al., 2014; Yu et al., 2014). Since BAP1 is recruited to DSBs together with its PcG protein partner ASLX (Ismail et al., 2014), one potential function of the PR-DUB might be regulation of (PcG-mediated) transcriptional silencing at DSBs (Shanbhag et al., 2010; Kakarougkas et al., 2014; Ui et al., 2015). It will be interesting to further investigate the interplay between BAP1 and the PcG repressive complexes PRC1 and PRC2 at DBS.

Similarly to BRCA1-loss, BAP1-deficient cells show hypersensitivity to PARP inhibition, a finding that suggests involvement of BAP1 in BRCA-mediated HR (Peña-Llopis et al., 2012; Ismail et al., 2014; Yu et al., 2014). In support of this hypothesis, Ismail et al. (2014) and Yu et al. (2014) reported reduced recruitment of BRCA1 and of key HR factors such as RAD51 and RPA at damage sites, concomitant with defective HR. The cellular interplay between BAP1 and BRCA1 is, however, far from being understood. On one hand, BAP1 was first discovered in a yeast two-hybrid screen as a BRCA1-interacting protein (Jensen et al., 1998). On the other, proteomic studies in human/mouse cells did not detect BAP1 in stable complex with BRCA1 (Sowa et al., 2009; Bott et al., 2011; Dey et al., 2012). *In vitro*, BAP1 antagonizes the ub ligase activity of the BRCA1/BARD1 complex (Nishikawa et al., 2009), this being the E3 complex with DDR functions (Greenberg et al., 2006). If this would hold *in vivo*, BAP1 may be important to regulate BRCA1/BARD1 activity and/or it may target putative/yet-undefined BRCA1 substrates during DDR (Kalb et al., 2014). Identifications of BAP1 targets during DDR will aid in clarifying whether BAP1-mediated regulation of BRCA1 activity is a mechanism to control HR.

Further, connecting BAP1 to PARP, it is of note that recruitment of GFP-BAP1 to laser-induced DSBs requires PARP activity, beside RNF8/RNF168 (Ismail et al., 2014). However, since BAP1 does not seem to bind directly to poly(ADP) ribose (PAR) polymers, PARP effect on BAP1 in DDR has been suggested to be indirect (Ismail et al., 2014). PARP has been reported to promote RNF168-mediated DDR signaling and repair (Smeenk et al., 2013), as well as it has been implicated in favoring recruitment of PcG proteins (MEL18 and CBX4) to DSBs (Chou et al., 2010). Also, the RNF8-related E3 CHFR, which appears to regulate the first wave of ubiquitination, requires PAR for its rapid recruitment to DSBs (Liu et al., 2013). Most likely, the link between PARP and BAP1 in DDR is among one of these activities.

### MYSM1/2A-DUB

The metalloprotease MYSM1 (also known as 2A-DUB) deubiquitinates uH2A *in vitro* and in cell lines (Zhu et al., 2007). Although it was originally identified as a transcriptional co-activator (Zhu et al., 2007), recent findings connect this DUB to DDR. MYSM1 has a distinctive domain architecture among the DUBs, harboring a SANT and SWIRM domains, which are frequently found in transcription factors and in DNA/chromatin-associated proteins (Boyer et al., 2004; Yoneyama et al., 2007). Consistently, ChIP assays detected enrichment of MYSM1 at numerous gene promoters (Zhu et al., 2007; Jiang et al., 2011; Wang et al., 2013). Interestingly, MYSM1 is also capable of binding to DSBs flanking chromatin, where it may favor repair (Nishi et al., 2014). Further linking MYSM1 to DDR, MYSM1 was identified as an ATM phosphorylation target upon damage (Matsuoka et al., 2007) and its deletion in mice confers sensitivity to total body IR (Wang et al., 2013). Spontaneous DNA damage may also be enhanced upon MYSM1 loss, as measured by increased γH2AX staining, micronuclei and oxidative stress in hematopoietic populations of MYSM-deficient mice (Nijnik et al., 2012; Gatzka et al., 2015). Yet, MYSM1 involvement in DDR and more specifically in the RNF8 pathway remains largely unexplored. MYSM1 activity has been mostly linked to the repressive mark H2AK119ub (Zhu et al., 2007; Jiang et al., 2011; Wang et al., 2013; Won et al., 2014), opening the possibility of MYSM1 participation in regulating DSB-induced gene silencing. Also, rather than targeting the DDR H2AK13/15ub mark (Mosbech et al., 2013), MYSM1 may, similarly to other JAMM/MPN+ metalloproteases (see below), display specificity for Lys63-linked ub chains (Komander et al., 2009), which are key DDR signals.

Finally, with regards to H2A DUBs, another DUB that has been reported to oppose to H2AX ubiquitination, thereby preventing recruitment of both 53BP1 and BRCA1 to DSBs is **DUB3** (Delgado-Diaz et al., 2014). At least five additional DUBs can limit RNF8-RNF168-mediated chromatin ubiquitination. These DUBs are more extensively reviewed elsewhere (Jackson and Durocher, 2013; Panier and Durocher, 2013). Among these, BRCC36 (BRCC3), POH1/PSMD14 and OTUB1 directly target ub conjugates on chromatin. The JAMM/MPN(+) DUBs **BRCC36 (BRCC3**; Dong et al., 2003; Sobhian et al., 2007; Shao et al., 2009) and **POH1/PSMD14** (Butler et al., 2012) act as negative regulators by displaying selectivity for K63-ub at DSBs. BRCC36 is part of the BRCA1-A complex and, together with Rap80, has been associated with inhibition of HR early upon DSB induction (Coleman and Greenberg, 2011; Hu et al., 2011). On the other hand, one of the reported functions of the 19S proteasome subunit POH1 is to promote HR, supporting contribution of DUB activity to DSB repair pathway choice (Butler et al., 2012; Kakarougkas et al., 2013). Further in line with such hypothesis, depletion of the otubain family DUB **OTUB2** resulted in decreased HR, as measured by the DR-GFP HR reporter assay (Kato et al., 2014). OTUB2 is thought to act early after damage by preventing RNF8-mediated ubiquitination of L3MBTL1, which proteasomal degradation is required for 53BP1 recruitment (Acs et al., 2011), as well as K63-ub (Kato et al., 2014). However, unlike for BRCC36, these K63-ub-conjugates do not include H2A, indicating the ability of these DUBs to target differential substrates (Shao et al., 2009; Kato et al., 2014). Notably, the DUBs OTUB1 and USP34 regulate DDR through indirect mechanisms. In fact, **OTUB1** attenuates ub-based DDR through non-catalytic inhibition of RNF168 activity (Nakada et al., 2010). Instead, **USP34** acts on RNF168 by removing degradative ub chains, thereby stabilizing the E3 and promoting DDR signaling (Sy et al., 2013). Also, indirect effects may underlie **USP11** regulatory activity in HR (Wiltshire et al., 2010). Finally, **USP28** is one more USP recruited via 53BP1 to DSBs. However, loss of USP28 did not cause significant DDR defects nor an altered phenotype in mice, indicating that this DUB does not have a prominent role in the DSB response (Knobel et al., 2014).

The substrate selectivity of the DUBs is critical is regulating DDR, given the diversity of ub signals at DSBs. Yet, how the DSB-associated H2A DUBs reach selectivity for H2AK119ub or H2AK13/15ub and if any of these DUBs can specifically oppose the DSB-induced H2AK13/15ub mark is still an open question. USP3 and USP44 potent activity toward steady state uH2A and their ability to limit 53BP1 IRIFs suggest that these DUBs can potentially target both the PcG-specific K119ub as well as the DDR-mediated K13/15ub marks on H2A (Nicassio et al., 2007; Doil et al., 2009; Mosbech et al., 2013; Sharma et al., 2014), the latter being a prerequisite for 53BP1 binding at damage sites (Fradet-Turcotte et al., 2013). Instead, USP16, BAP1 and MYSM1, lacking clear impact on 53BP1 IRIFs (Mosbech et al., 2013), may not be directly involved in H2AK13/15ub deubiquitination.

Notably, chromatin states are expected to impact on the recruitment/activity of the DUBs. In fact, MYSM1, which is found in complex with the histone acetyl transferase p/CAF, has been reported to be more active toward hyperacetylated nucleosomal substrates (Zhu et al., 2007). Also, recent ChIP-sequencing approaches indicate that active transcription marks, such as H3K36Me3, specify the recruitment of HR proteins to DSBs (Aymard et al., 2014), and readers of acetylated chromatin (bromodomain containing proteins) re-localize to DNA damage sites to promote gene silencing and repair by HR (Gong et al., 2015).

As to the “ubiquitin code,” it is likely that, besides K48-, K63- and the less characterized K6-linked ub chains (Morris and Solomon, 2004), additional “atypical” ub polymers (Kulathu and Komander, 2012) participate in DSB signaling. Such examples are the recently reported K27-linked ub chains catalyzed by RNF168 on H2A/H2AX (Gatti et al., 2015). Also, K27-ub might be relevant for PcG-mediated DDR, as auto-ubiquitination of RING1B through mixed polyub chains (K6-K27-K48) is a prerequisite for its ability to mono-ubiquitinate H2A *in vitro* (Ben-Saadon et al., 2006). While a few DDR DUBs display ub-linkage selectivity, such as the JAMM proteases BRCC36 and POH1 for K63-ub, OTUB1 for K48-ub and OTUB2 for K63-ub (Butler et al., 2012; Komander and Rape, 2012; Mevissen et al., 2013; Kato et al., 2014), the ub-linkage selectivity of many other (DDR) DUBs is unclear. Potentially, DUBs belonging to the USP family can hydrolyze all linkages, al least when synthetic ub dimers are used as substrates *in vitro* (Faesen et al., 2011). Elucidation of DUB selectivity/regulation of activity in DDR awaits dedicated cell-based as well as *in vitro* assays, for example employing relevant substrates such as nucleosomal particles and (DDR-) specific E3/E2 ligases.

Finally, the ability to deubiquitinate multiple substrates is common within DUBs and the low degree of selectivity shown by the USPs *in vitro* suggests that other mechanisms rather than molecular substrate selection (i.e., regulation by cofactors, post-translational modifications, subcellular localization, cell cycle regulation) may have a greater role in determining their specificity *in vivo* (Komander et al., 2009; Clague et al., 2012; Sahtoe and Sixma, 2015). Indeed, for instance, BAP1 fails to deubiquitinate H2A if not in the context of the PR-DUB (Scheuermann et al., 2010). Similarly, the JAMM proteases POH1 and BRCC36 require protein–protein interactions for DUB activity and BRCC36 is targeted in the nucleus to DSBs or to cytoplasmatic functions by differential association with RAP80 or SHMT respectively (Cooper et al., 2009; Zheng et al., 2013). The use of catalytically inactive mutants that allows to circumvent the transient nature of the DUB/substrate interaction (Nicassio et al., 2007; Sowa et al., 2009), and conditions of DNA damage in proteomic approaches may help toward the identification of DUB targets/cofactors relevant for DSB signaling.

## DSBs-Associated DUBs: Implications in Stem Cell Maintenance and in Cancer

De-regulation of DDR mechanisms can contribute to cancer but may also promote functional decline of the stem cells with consequential deterioration in tissue function and aging (Jackson and Bartek, 2009; Blanpain et al., 2011; Behrens et al., 2014). Consistent with this, recent studies uncovered the relevance of DSB-associated DUBs in preserving tissue function. Here, I will focus on the emerging roles of the H2A DUBs USP3, USP16, USP44, BAP1, and MYSM1 in HSC maintenance and cancer. The direct investigation of the consequences of inactivation of some of these DUBs has been so far restricted to HSCs for practical reasons. However, the recent advances in our understanding of stem cell niches in several organs warrant extending these studies to other tissues, which might uncover unique dependencies for individual components of the pathway.

### DSBs-Associated DUBs in Hematopoietic Stem Cell Biology

Hematopoietic stem cells maintain homeostasis and replenish the blood system throughout life, by their ability to self-renew. DNA damage accumulates in HSCs during aging in mice and in man (Rossi et al., 2007; Rübe et al., 2011; Beerman et al., 2014), and it has become clear that genome repair is important for the HSC regenerative potential. In fact, DNA damage to the HSC pool has been identified as an underlying cause of BM failure in patients suffering from FA, an inherited DNA repair deficiency syndrome (Ceccaldi et al., 2012). In particular, a strong p53 response to replication stress and unresolved DNA damage was characterized as a critical mechanism for the progressive loss of HSCs and hence BM failure in FA patients (Ceccaldi et al., 2012). Further supporting a crucial role of DDR in HSC homeostasis, mouse models with engineered mutations in different DNA repair and DDR genes, besides FA related genes, manifest severe hematopoietic phenotypes and HSC deficiencies, in particular under conditions of stress (Ito et al., 2004; Nijnik et al., 2007; Rossi et al., 2007; Niedernhofer, 2008; Garaycoechea et al., 2012; Wang et al., 2012; Walter et al., 2015).

DNA damage, such as damage arising from inefficient DNA replication or from reactive oxygen species (ROS) and DSBs, can have two major consequences in HSCs. First, unrepaired DSBs may drive HSCs cell cycle arrest, senescence or apoptosis, or premature differentiation leading to their loss of function and consequent aging phenotypes and organ failure (Wang et al., 2012; Flach et al., 2014). Second, the accumulation of mutations providing selective advantage to the mutated cells can lead to cancer, as exemplified in myelodysplastic syndrome (MDS) and myelogenous leukemias (Blanpain et al., 2011; Behrens et al., 2014). Notably, the consequences of chronic genotoxic stress/persistent DNA damage signaling are mostly evident in tissues with high cell turnover and relying upon expansion of a limited number of stem/precursors cells, such as the hematopoietic system (Bender, 2002; Rossi et al., 2007). This makes the hematopoietic system potentially sensitive to even modest alterations in the ub-equilibrium in homeostasis as well as during ub-mediated DDR. An example in this direction is given by deletion of the DUB USP1 in mice. USP1 is a regulator of DNA-interstrand cross-links repair by the FA pathway through deubiquitination of one of its critical factor at the chromatin, FANCD2 (Kim et al., 2010). Consistent with such a role, USP1 deletion reduces the repopulation abilities of mouse HSCs (Parmar et al., 2010).

A link between the ub-based response to DSBs and HSC maintenance emerged from recent *in vivo* studies on **USP3**. Indeed, a clear pathological manifestation of *Usp3* deletion in mice is progressive lymphopenia upon aging (Lancini et al., 2014). Such an altered lineage potential and immunodeficiency occur in the elderly and it has been linked to an impaired functional capacity of the aged HSCs (Geiger et al., 2013). Consistently with an age-dependent cellular attrition, USP3-deficient HSCs displayed a marked decline in cell number and activity over time, as demonstrated in BM transplantation experiments (Lancini et al., 2014). Is this phenotype related to DNA damage? In support of this hypothesis, we found that USP3-deficient HSCs accumulate spontaneous DNA damage, are hypersensitive to IR *in vivo* and inefficiently resolve 53BP1 IRIFs and DSBs *in vitro* (Lancini et al., 2014). Collectively, these data suggest a role for USP3 in protecting the HSCs from DNA damage by restraining the ub-dependent DDR pathway (Lancini et al., 2014). Yet, the source of spontaneous DNA damage measured in USP3-deficient HSCs has not been identified. In absence of direct evidences, replication-associated genotoxic stress, which is thought to potently contribute to normal HSC decline during aging (Flach et al., 2014), may certainly be an accountable one. In fact, young USP3 knockout BM performed poorly in serial transplantations, a situation of enforced proliferation resembling what happens during aging (Lancini et al., 2014).

In line with an involvement of the RNF8-RNF168 pathway in hematopoiesis, inactivating mutations in RNF168 are associated with the RIDDLE syndrome, characterized by cellular radiosensitivity and immunodeficiency (Stewart et al., 2009), features which are recapitulated in knockout mouse models for these E3s (Li et al., 2010; Santos et al., 2010; Bohgaki et al., 2011). Whether RNF8/RNF168 deficiency also impact on HSC maintenance has not been reported, yet significant reduction of BM cellularity was measured upon RNF8 loss (Li et al., 2010). USP3 as well as RNF8 and RNF168-deficient mice constitute valuable *in vivo* models to gain insights into how lack of control of the ub-dependent DDR pathway may contribute to the functional decline observed in aged HSCs.

The ***Usp16*** gene is located on human chromosome 21, a chromosome that is triplicated in Down’s syndrome. Trisomy of *Usp16* was recently associated with reduced HSC self-renewal in a mouse model for Down’s syndrome (Ts65Dn), whose HSCs express 1.5-fold higher levels of *Usp16* mRNA than wild type HSCs (Adorno et al., 2013). USP16 can deubiquitinate H2AK119ub (Joo et al., 2007), a critical mark for epigenetic control of stem-cell identity/maintenance and differentiation by the PcG E3 RING1B/BMI1 (Sparmann and van Lohuizen, 2006; Di Croce and Helin, 2013). Relevant to this review, BMI1 is essential to HSC self-renewal through mechanisms involving repression of the *Cdkn2a* tumor suppressor locus as well as protection from oxidative stress and DNA damage (Lessard and Sauvageau, 2003; Park et al., 2003; Liu et al., 2009). Notably, Adorno et al. (2013) linked increased *Usp16* gene dosage with reduced H2A ubiquitination at the *Cdkn2a* locus and with a concomitant increase in senescence of Ts65Dn fibroblasts. This suggests that *Usp16* trisomy contributes, at least in part, to the cellular defects of Ts65Dn mice through modulation of the BMI1 self-renewal and/or senescence pathway (Adorno et al., 2013). Increased ROS and markers of oxidative stress were also reported in Ts65Dn hematopoietic stem and progenitor cells (HSPCs) (Lorenzo et al., 2011). It will be interesting to investigate whether *Usp16* trisomy contributes to this phenotype and whether Ts65Dn HSCs/fibroblasts suffer from augmented DNA damage/ DDR defects.

**MYSM1**, first linked to epigenetic control of B-cell development in mice (Jiang et al., 2011), recently emerged as a critical regulator of hematopoiesis. Notably, beside distinct multi-lineage defects in hematopoietic cell differentiation (Jiang et al., 2011; Nijnik et al., 2012; Nandakumar et al., 2013; Won et al., 2014), loss of MYSM1 in mice results in severe reduction of the HSC pool size and impairment in their *in vivo* repopulation capacity (Nijnik et al., 2012; Wang et al., 2013). While relative limited transcriptional changes were detected in *Mysm1*-null compared to wild type HSPCs, Gfi1, one of the critical HSC factors, was identified as potential target for MYSM1-mediated epigenetic regulation (Wang et al., 2013). A consistent feature of *Mysm1*-null HSPCs that likely contributes to their reduced cellularity is an elevated apoptosis rate, accompanied by elevated ROS and γH2AX (Nijnik et al., 2012; Wang et al., 2013; Gatzka et al., 2015). Oxidative stress, accumulation of DNA damage and p53 activation are frequently associated with HSC deficiency and BM failure syndromes (Ito et al., 2004; Niedernhofer, 2008; Liu et al., 2009; Ceccaldi et al., 2012). Consistently, upregulation of p53 and of pro-apoptotic p53 target genes was measured in MYSM1-deficient cells (Nijnik et al., 2012; Belle et al., 2015; Gatzka et al., 2015) and p53 co-deletion significantly rescued HSCs numbers and activity in *Mysm1*-/-; *p53*-/- double mutant mice (Belle et al., 2015; Gatzka et al., 2015). As mentioned before, BMI1 plays a crucial role in protecting mouse cells, including HSCs, from mitochondrial dysfunction, ROS and DNA damage (Liu et al., 2009). Intriguingly, however, while antioxidants could rescue *Bmi1*-/- mice (Liu et al., 2009), similar treatments did not rescue *Mysm1*-/- mice developmental and hematopoietic phenotypes, suggesting that mechanisms other that oxidative stress may contribute to p53 activation in the context of MYSM1 loss (Belle et al., 2015). Further investigation of such p53-inducing cellular stresses and of MYSM1 links with DDR merits further studies. Perhaps relevant in this regard, Wang et al. (2013) reported exit from homeostatic quiescence for *Mysm1*-/- HSCs, a condition recently suggested to induce DNA damage and attrition in HSCs upon a range of physiological stresses (Walter et al., 2015). Notably, a homozygous truncating mutation in the *MYSM1* gene was reported in two young siblings suffering form anemia, mild thrombocytopenia and lymphopenia, underlying its relevance in inherited BM failure disorders (Alsultan et al., 2013). Finally, induction and repair of programmed DSBs by NHEJ is a physiological process crucial for immune cells development (Alt et al., 2013). Whether DSB repair defects contribute to the developmental deficiencies reported in MYSM1-deficient B and T cells (Jiang et al., 2011; Nijnik et al., 2012; Gatzka et al., 2015) represent an interesting question for future investigation.

Dey et al. (2012) uncovered a novel role of **BAP1** in HSC homeostasis. Indeed, BAP1 is expressed in the murine HSPC compartment and its deletion in adulthood resulted in skewing of differentiation toward the myeloid lineage and decreased HSC survival/self-renewal. BAP1 involvement in HSC function is intriguing and the effect of BAP1 loss on HSC quiescence, cell cycle progression and proliferative capacity warrant further investigation. While several genes including regulators of hematopoietic cell survival were identified as potential BAP1 targets in mouse HSPCs (Dey et al., 2012), it will be informative to address whether BAP1-deficient HSCs experience exacerbated DNA replication stress/DNA damage and/or DDR defects, which might contribute to their functional decline.

### DSBs-Associated DUBs in Cancer

The importance of some of the previously mentioned factors in human cancer (e.g., BRCA1, 53BP1) underscores the impact of this pathway on human health (Jackson and Bartek, 2009; Gudjonsson et al., 2012). The analysis of RNF8- and RNF168-deficient mice further supports a role of these key E3 ligases in tumor suppression (Li et al., 2010; Bohgaki et al., 2011). Notably, analogously to RNF8, ***Usp3***-deleted mice develop a broad spectrum of tumor types with a latency of 1 year of age (Lancini et al., 2014). Such broad spontaneous neoplasia in USP3-deficient mice might be linked to enhanced genomic instability, as observed in primary MEFs form these animals (Lancini et al., 2014). Further, **MYSM1**-deficient mice are tumor prone, developing thymic lymphoma with a latency of 4–6 months (Belle et al., 2015). Deletion of ***Usp16*** in mice is instead early embryonic lethal and the consequences of its inactivation in adult tissues have not been explored (Yang et al., 2014). I will next focus on USP44 and BAP1, which have been best characterized as tumor suppressors in human pathology.

**USP44** knockout mice are prone to develop spontaneous tumors, displaying in particular an approximately nine-fold increase in adenomas of the lung compared to wild type upon aging (Zhang et al., 2012). Notably, *USP44* was also found frequently down-regulated in human bronchial adenocarcinomas and patients with low *USP44* expression had significantly shorter overall survival, underscoring a tumor suppressive function in human cancer (Zhang et al., 2012). Zhang et al. (2012) reported that mouse cells lacking USP44 not only show a defect in silencing the mitotic checkpoint, a flaw linked to its deubiquitination activity toward the APC activating subunit CDC20 (Stegmeier et al., 2007), but they mis-segregate their chromosomes and exhibit whole chromosome aneuploidy (Zhang et al., 2012). These findings suggest that one mechanism by which USP44 suppresses tumorigenesis is by preventing aneuploidy, a feature commonly associated with human cancer (Zhang et al., 2012). Given USP44 novel implication in the control of DSB-induced chromatin ubiquitination, it is plausible that DDR defects may contribute to genomic instability and to the tumor prone phenotype of *Usp44*-/- mice (Mosbech et al., 2013). Moreover, USP44 has been reported to regulate gene expression and to prevent embryonic stem cell differentiation through H2B deubiquitination (Fuchs et al., 2012). It will be important to determine which of the functions of USP44 are critically required for tumor suppression.

**BAP1** is an established tumor suppressor, with its genomic locus being frequently deleted in human cancer. In particular, prevalent somatic and germline *BAP1* loss/inactivating mutations are found in metastatic uveal melanomas, malignant pleural mesothelioma and renal cell carcinomas (Harbour et al., 2010; Bott et al., 2011; Peña-Llopis et al., 2012; Carbone et al., 2013). The recent studies by Dey et al. (2012) revealed a potent tumor suppressive function of BAP1 also in myeloid neoplasia. This is supported by the observation that specific loss of *Bap1* in mouse hematopoietic progenitors is sufficient for the propagation of features of human MDS upon transplantation and by the identification of *de novo BAP1* mutations in MDS patients (Dey et al., 2012). The exact mechanism through which BAP1 regulates cell proliferation and tumorigenesis is not known and deciphering BAP1 functions is complicated by the diversity of protein (complexes) BAP1 interacts with in mammalian cells, these including PcG repressors (Bott et al., 2011; Dey et al., 2012; Peña-Llopis et al., 2012). Collectively, however, the current data based on quantitative analysis of BAP1 protein interactions and target genes identification implicate transcriptional de-regulation in the pathogenesis of BAP1-associated mesothelioma, renal cancer and myeloid neoplasia (Bott et al., 2011; Dey et al., 2012; Peña-Llopis et al., 2012). Consistently with a BAP1/PR-DUB functional role in PcG-mediated repression in *Drosophila* (Scheuermann et al., 2010), the complex BAP1-associated transcriptional network in mammals includes expression programs regulated by PcG proteins (Bott et al., 2011; Dey et al., 2012; Peña-Llopis et al., 2012), the genetic alteration of which predisposes to the development of various cancers (Sparmann and van Lohuizen, 2006; Di Croce and Helin, 2013).

As previously discussed, BAP1-deficient cancer cell lines were shown to be more sensitive to IR and to PARP inhibitors (Peña-Llopis et al., 2012; Ismail et al., 2014). Restoration with wild type BAP1, but not with catalytically inactive BAP1 or with mutant BAP1 mimicking cancer-associated mutations, protects the cells against genotoxic death (Peña-Llopis et al., 2012; Ismail et al., 2014). These studies indicates faults in DDR upon BAP1 loss and provide a platform to further investigate how (cancer-)inactivating mutations may affect BAP1-mediated DDR signaling/DSB repair and thereby contribute to tumor suppression. Of note, BAP1 is a substrate of the ATM/ATR checkpoint kinases, and it is phosphorylated upon several stressors, including IR, UV light and replication stress (Matsuoka et al., 2007; Eletr et al., 2013; Ismail et al., 2014; Yu et al., 2014). Therefore, pleotropic effects of BAP1 in DDR pathways can be expected.

Given the multiple roles of uH2A, the consequences of deregulation of H2A targeting DUBs *in vivo* are complex and the field is confronted with the challenge of dissecting the molecular mechanisms involved. USP3 has been mainly linked to the DDR and does not appear to have a prominent role in gene expression (Lancini et al., 2014). Instead, while it is clear that USP16, BAP1, and MYSM1 can regulate gene transcription of, among others, PcG targets (Joo et al., 2007; Bott et al., 2011; Dey et al., 2012; Nijnik et al., 2012; Peña-Llopis et al., 2012; Adorno et al., 2013; Wang et al., 2013; Yang et al., 2014), the involvement of these DUBs in DDR has just emerged and the mechanism by which they contribute to genome stability is an important area for future studies.

Another critical question is the relevance of DDR-DUBs for human cancer. As discussed earlier, BAP1 is an established tumor suppressor in man. Also, Dub3 has oncogenic potential, a function that has been primarily related to its ability to regulate the crucial cell cycle regulator Cdc25A phosphatase (Pereg et al., 2010). As to the other DUBs discussed in this Review, cross-cancer genetic alterations of the H2A-DUBs USP3, USP16, USP44 and MYSM1, as well as for BRCC36, OTUB1, OTUB2 have been reported (cBio Portal for cancer genomics, http://www.cbioportal.org/index.do; Dong et al., 2003). However, the role of these DUBs in human cancer remains to be elucidated.

Finally, RNF8/RNF168 amplification is frequently found in human tumors (TCGA, 2012) and RNF168 gain of function was connected with human papillomavirus (HPV)-positive cervical cancers (Gudjonsson et al., 2012). Lack of control of ub-mediated DDR signaling is a threat to genome integrity, and so potentially oncogenic, for instance during mitosis (Orthwein et al., 2014) and at telomeres, where the RNF8 pathway promotes illegitimate repair of chromosome ends (Peuscher and Jacobs, 2011). Notably, the role of DUBs in physiological inhibition of the RNF8 pathway at telomeres is emerging, with BRCC36 opposing to RNF168 to suppress chromosome end-to-end fusions (Okamoto et al., 2013).

## Concluding Remarks

While it is clear that DUBs are critical regulators of the DSB response, we are only beginning to understand their molecular mechanisms of action, the consequences of their deregulation on genomic stability and their impact on organism physiology and human disease. A key unresolved question is the functional and molecular interplay between the DUBs in determining the ub-DDR cascade. Indeed, cooperation between DUBs-regulated functions likely occurs, and given the potential of functional compensation *in vivo*, such crosstalk is critical for genome maintenance and cell fate outcomes (Gudjonsson et al., 2012; Mosbech et al., 2013; Lancini et al., 2014; Nishi et al., 2014). Small-molecule inhibitors to DUBs are being developed with important achievements (Ernst et al., 2013; Jacq et al., 2013; Zhang and Sidhu, 2014). Gaining insights into DUBs functional roles and molecular networks in the DSB response could provide novel rationale for pursuing dedicated DUBs as drug targets.

## Conflict of Interest Statement

The author declares that the research was conducted in the absence of any commercial or financial relationships that could be construed as a potential conflict of interest.

## Abbreviations

BM: bone marrow
FA: Fanconi anemia
HR: homologous recombination
HSC: hematopoietic stem cell
HSPC: hematopoietic stem and progenitor cell
IRIFs: ionizing radiation induced foci
K63-ub: lysine 63-linked ubiquitin chains
PAR: poly(ADP-ribose)
PARP: poly(ADP-ribose) polymerase
PcG: polycomb group protein
ub: ubiquitin
uH2A: mono-ubiquitinated histone H2A
uH2B: mono-ubiquitinated histone H2B.
